# A Kinship-Based Modification of the Armitage Trend Test to Address Hidden Population Structure and Small Differential Genotyping Errors

**DOI:** 10.1371/journal.pone.0005825

**Published:** 2009-06-08

**Authors:** Cyril S. Rakovski, Daniel O. Stram

**Affiliations:** 1 Department of Mathematics and Computer Science, Chapman University, Orange, California, United States of America; 2 Department of Preventive Medicine, University of Southern California, Los Angeles, California, United States of America; Erasmus MC, Netherlands

## Abstract

**Background/Aims:**

We propose a modification of the well-known Armitage trend test to address the problems associated with hidden population structure and hidden relatedness in genome-wide case-control association studies.

**Methods:**

The new test adopts beneficial traits from three existing testing strategies: the principal components, mixed model, and genomic control while avoiding some of their disadvantageous characteristics, such as the tendency of the principal components method to over-correct in certain situations or the failure of the genomic control approach to reorder the adjusted tests based on their degree of alignment with the underlying hidden structure. The new procedure is based on Gauss-Markov estimators derived from a straightforward linear model with an imposed variance structure proportional to an empirical relatedness matrix. Lastly, conceptual and analytical similarities to and distinctions from other approaches are emphasized throughout.

**Results:**

Our simulations show that the power performance of the proposed test is quite promising compared to the considered competing strategies. The power gains are especially large when small differential differences between cases and controls are present; a likely scenario when public controls are used in multiple studies.

**Conclusion:**

The proposed modified approach attains high power more consistently than that of the existing commonly implemented tests. Its performance improvement is most apparent when small but detectable systematic differences between cases and controls exist.

## Introduction

In concept, the methods for analysis of case-control data with regard to association between potential risk factors and the probability of an event of interest are based on assessment of the differences in covariate distributions between cases and controls. However, the presence of such systematic differences could be attributable to hidden unaccounted factors; that will result in greatly inflated type I error rates as multiple tests are applied to the data. Even though similar scenarios occur with environmental and other common risk factors, the problem of analyzing case-control data with inherent distributional differences is most readily seen in genome-wide association scans. In these settings, the systematic differences are driven by complex hidden population structure and relatedness and the resulting increased rate of false-positive individual marker tests can completely obfuscate the signal from the true causal gene.

There have been several approaches that attempt to provide solutions to this problem, for a recent comprehensive review see [1]. The simplest but indirect way of addressing the effect of hidden structure is to assess its effect on a random set of markers tests and adjust all statistics by a common scaling factor chosen in way that guarantees an adherence to the nominal type I error rates [2]. However, the method is susceptible to power deficiency since the implementation of the genomic control adjustment fails to change the significance levels order of the analyzed tests. Other more precise and direct methods involve actual modeling of the underlying population complexity. Structured association [3] is an alternative method that uses random SNPs to infer the hidden population structure expressed through a matrix *Q*. This information is incorporated in the subsequent stratified analysis and its implementation results in desirable type I error rates and improved power. This method has recently been implemented in a computationally efficient way that addresses the intensity of whole genome scans [4] but is highly sensitive to the way the elements of *Q* are defined. The principal components approach modifies the classical Armitage test for trend by adjusting the genotypes with respect to the major axes of variation (eigenvectors) of the empirical variance-covariance matrix [5]. Thus, this method subtracts approximate effects of the underlying population structure from the original genotypes and the resulting adjusted genotypes are considered to contain only the true associations. It has been shown that principal components method possesses a moderate power advantage over the genomic control. However, the principal components approach can fail to recognize and consequently adjust for complex population structures [6]. Further, a related generalized linear model approach has recently been shown to provide an improved stratification correction [7].

Another commonly used approach for modeling correlated quantitative trait observations while adjusting for population structure is the mixed model [8]. It requires separate modeling of the mean and the variance structure and both models reflect an assessment of the detectable population structure. The model for the mean incorporates a population structure matrix *Q* (this is the same matrix used in structured association) and an explicit design of the variance as a function of a kinship matrix *K* that reflects the relatedness between all pairs of subjects. In work described by [6] the mixed model approach provided improved type I error rates and higher power compared to principal components and structured association, in a highly structured example. However, the complexity of the mixed model algorithms raise questions about their practicality in the whole genome setting. Furthermore, practical implementations of the mixed model extension to appropriately address case-control data are not straightforward.

In this work we propose a method that combines advantageous features of genomic control, principal components, and mixed model strategies that reflects the idea that adjusting for a well-defined variance covariance structure in a linear model with a simple mean structure is the optimal testing strategy. Further, we show that the new approach possesses a distinct advantage in addressing a potential issue related to the use of public controls for multiple associations studies that can result in the presence of differential DNA preparation-related differences between cases and controls [9]. We show that the effect of even small DNA preparation differences adversely affects the performance of the principal components method by inducing overcorrection and a consequent decrease in power.

## Methods

In case-control settings, testing strategies are usually based on modeling the dichotomous affection status outcome variable. Instead, we propose a series of models for the vectors of allele counts at each SNP; we adopt the classical theory of linear models for non-identically distributed outcome variables that follow an unspecified general distribution [10].

Let 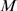 and 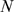 be the number of SNPs and 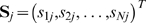, 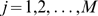 denote the vector of genotypes at the 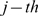 marker for all N subjects. We assume that 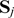 follows an unspecified multivariate distribution with mean vector ***μ***_*j*_ (of length N) and an (N×N) variance-covariance matrix 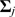.

For example, consider the covariance matrix for 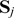 that is imposed by the well-known beta-binomial model of Balding and Nichols [11] which is often used to study the effects of hidden structure [5], [8]. This approach explicitly models current day populations via their divergence from an ancestral population specified by Wright's *F_st_* statistic. If there are *L* such populations 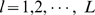 with corresponding divergence coefficients 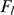, the covariance matrix for any marker **S** *_j_* sampled from this model can readily be shown to be of form 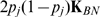 in which *p_j_* is the frequency in the ancestral population of marker *j* and **K** ***_BN_*** is a fixed matrix (for all markers) with diagonal terms equal to 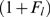 and off-diagonal terms equal to either zero (for subjects in different modern-day populations) or 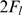 for subjects in the same populations. Thus, the correlation between 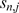 and 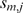 for subjects *n* and *m* who are both members of the 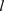 *th* modern day population will equal 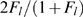. In case-control studies simulated using this model the true distribution of 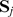 is complex and generally unknown due to hidden structure, hidden relatedness and unknown SNP function.

Notice that in the Balding-Nichols model the matrix **K** *_BN_* is common for all markers, which suggests estimating **K** *_BN_* using rescaled genotype vectors 
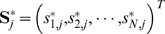
 with 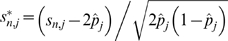. If 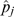 is a consistent estimate of the ancestral population allele frequency of the *j* th marker then each of these 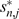 will have (approximately) constant variance 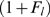 and correlations between 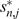 and 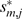 either zero (for members of different populations) or equal to 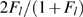 (for members of the 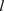
th hidden population). Thus, a natural estimate of **K** *_BN_* will be the average of the outer products of 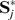, 
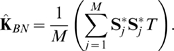



Unfortunately, this estimator is unrealizable in practice as it is unlikely to obtain a consistent estimate for the ancestral population allele frequency 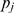 at each locus and therefore, one generally substitutes ½ the sample mean 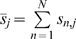 for 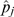 in the above-mentioned expression. Actually, the same estimator was used by Price et al.[5] for estimation of principal components. Clearly, this substitution would be expected to have some deleterious effect on our ability to estimate **K** *_BN_* and we explore this issue in our simulations below.

Specifically, we propose the following model for the variance structure: 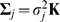, where 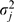 is the variance of the 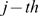 marker in the pooled sample and **K** is an appropriately chosen kinship matrix reflecting the relatedness between pairs of subjects. In our notation **K** is a relatedness matrix of unknown form (to be estimated from the full complement of genotype data) while **K** *_BN_* is a specialization of **K** to the Balding-Nichols model. In our analyses, we explored several versions of the kinship matrix **K** such as the implementation of the SPAGeDi software package [12], other identity by descent sharing based methods as well as the adjusted correlation matrix of Price et al. Our results show that there were not meaningful differences in our test performance when different versions of **K** were implemented. Following the last approach, the 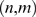 element of **K** is the adjusted correlation between the SNPs for subject *n* with the SNPs for subject *m* and is estimated as, 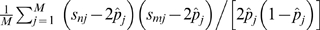, where 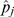 is the frequency estimate defined above.

However, unlike Price et al., we propose a simple model for the mean that avoids adjustment for the population structure in this part of the model. Let 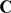 be a 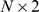 matrix containing a vector of ones and a vector of zeros and ones denoting case-control status and let 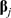 denote a parameter vector with two elements (*β_1j_,β_2j_*)^T^. We implement the following model for the mean of the *j-th* SNP, 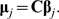
(1)Here 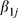 is (twice) the allele frequency of the 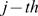 SNP for the controls and 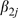 is (twice) the difference between the allele frequencies for the cases and controls. Under this general model for each SNP, we compute the Gauss-Markov best linear unbiased (BLUE) estimates of 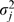 and 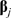, 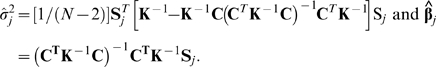
Then, the corresponding variance estimate for 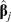 is given by,
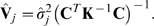
Finally, the chi-square test for association that we propose is given by,
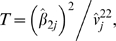
(2)where 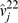 is the 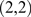 element of 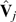. Note that when using the adjusted correlation matrix to estimate **K** the estimate is of rank at most 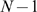 a consequence of the estimation of all allele frequencies *p_j_* from the combined data for cases and controls. Specifically, since the sum 
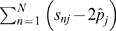
 equals zero for each SNP the first column of **C** (i.e. the vector of ones) is in the null space of **K** and 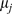 becomes non-estimable. Therefore, we must utilize a generalized inverse for **K** and 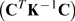 throughout when carrying out the necessary calculations. In fact, the latter matrix has just one nonzero element, namely the (2,2)th but this complication could be avoided by completely dropping 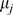 from model (1), removing the column of ones from **C**, and replacing 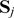 with 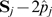 in the calculations. In our own computations, however, we simply utilized a standard generalized inverse (easily computed using the eigenvector/eigenvalue decomposition of K).

Note that this adjusted Armitage test is conceptually similar to the genomic control method. The new method uses a common adjusted correlation matrix for all SNPs which is analogous to the correction by genomic control of chi-square statistics by a common over-dispersion factor λ. In fact, **K** may be more properly thought of as an over-dispersion matrix than a correlation matrix since it does not necessarily have diagonal elements equal to 1. In the Balding-Nichols model with known ancestral allele frequencies it would have diagonal elements equal to the corresponding values of 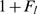. Unlike genomic control, the new test reorders as well as rescales the individual SNP statistics when structure is evident. There are also close connections among the Armitage trend test, principal components method and the modified Armitage test proposed here. The first is equivalent to a chi-square test derived from a linear model similar to the one used above with the distinction of assuming i.i.d. error structure so that **K** is an N×N identity matrix. The second is equivalent to a chi-square test derived from the linear model of the unadjusted Armitage test with the addition of the first few eigenvectors of **K** as covariates to the model for the mean.

We also note that the new test is closely related to the quasi-likelihood score (QLS) test of Bourgain et al. [13]. The authors propose a QLS test that also utilizes a model for **S** *_j_* in which the mean depends upon case-control status and with a covariance matrix for **S** *_j_* that is proportional to a fixed kinship matrix. Given this common starting point, it is not surprising that the resulting QLS estimators are in practice similar to the Gauss-Markov estimators described here. In Bourgain et al., the kinship matrix **K** is assumed to be known from first principles based upon known pedigree relationships among individuals. Our main innovation is the substitution for **K** of an empirical estimate based upon the availability of large scale genotyping data.

Next, we provide examples of the implementation of our method using specific data.

In certain cases our adjusted Armitage test corresponds to other well-known estimators. For example, consider a study in which siblings are used as controls in a 1-1 matched (discordant sibpair) design. In the special case of no hidden stratification the matrix **K** will be block diagonal with blocks equal to 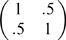. By carrying out the matrix calculations we see that the estimate 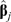 is equal to
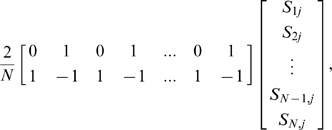
where subjects 1, 3, 5, …, N-1 are the cases and subjects 2, 4, 6, …, N are the controls. Similarly, the estimated variance matrix for 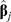 is easily shown to have (2, 2) element equal to 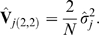 Thus, the test for *β_2,j_* = 0 will be just 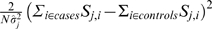 which is essentially the square of the paired t-test for the mean of the number of copies of marker *j* in the cases being equal to the mean of the number of copies of marker *j* in the controls.

Consider next the case of family trios (parents and an affected offspring) in the absence of hidden stratification and non-random mating. Here, **K** will be block diagonal with blocks equal to
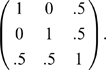
We code the offspring as “cases”, the parents as “controls” in the second column of **C** and obtain the estimator 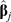 equal to 
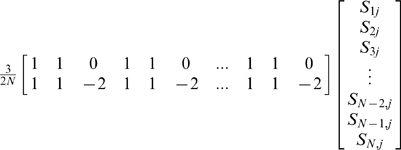
and its variance estimator equal to 
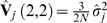
. Thus, the adjusted Armitage test is simply 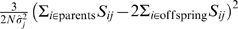 which compares the observed genotypes in the offspring to their expectations given the genotypes of the parents. The squared term in the numerator is the same as used in the TDT test. The variance term is slightly different, and can readily be shown to correspond to an estimate of the unconditional expectation of the conditional variances used in the TDT. Therefore, these tests will be asymptotically equivalent under the assumptions made above (i.e. that this **K** correctly captures the relatedness between subjects).

Next, we explore the performance of the new approach via simulations.

Initially, we conducted two simulation studies focused on the problem of dealing with distinct hidden population strata, one with and one without differential genotyping error between the cases and the controls. We simulated very severe population stratification for illustrative purposes. In our preliminary analyses we varied a multitude of parameters such as number of strata, distribution of cases and controls among strata, number of SNPs, risk model for the causal SNPs, and amount of DNA differentiation between cases and controls. Since the essence of our conclusions remained unchanged when these factors were repeatedly modified, we report results under the assumption that there were 10 equally sized populations and with baseline risks ratios 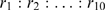 conforming to the following relations 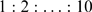. Therefore, mimicking the outcome of random sampling, the expected number of cases from the 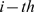 strata were assigned as 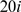. In order to speed up the simulations and as implemented in several other studies including Price et al.^1^, we generated SNPs conditional on case-control status (with genotype probabilities determined by true allele frequencies, baseline risk ratios, and a “rare-disease” assumption) rather than sample cases and controls from a large population. In a further simplification, we fixed the number of cases and controls from each subpopulation to be equal to their expected values (generating genotypes for exactly 110 controls and 20*i* cases from each). As mentioned, the results of the simulations do not depend to any meaningful extent on this simplification.

In this manner, we simulated genetic data consisting of 100,000 SNPs for a total of 1100 cases and 1100 controls under two different scenarios: with and without 1% random differential DNA preparation difference. This DNA differentiation was random and independently assigned to each SNP through a change to the allele frequencies of the cases but was not assigned for the controls. Thus, this was designed to reflect the scenario that could arise when using the same controls for multiple association studies with DNA from cases being prepared and/or genotyped using different methods than the controls. Specifically, we first drew an ancestral allele frequency 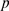 uniformly from 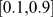 for each SNP. Then, under the Balding-Nichols model [11] for allele differentiation of distinct population strata as functions of the Wright's coefficients 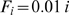, we drew the corresponding strata-specific allele frequencies from a Beta distribution with parameters 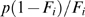 and 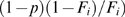.

Next, we set the relative risks of the 20 causal SNPs to be equal to 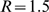 (as we found that the conclusions of the analyses are consistent with respect to the values of 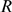), the relative risks of the non-causal SNPs to be equal to 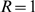 and computed the conditional distributions of the genotypes for the cases and controls for each population strata.

For the simulations under the second scenario, previous to the final step of generating genotypes for the non-causal SNPs for the cases, we changed their allele frequencies by a normal random variable with mean 0 and standard deviation 0.01 in order to represent “one percent” differential genotyping error between cases and controls.

In a natural continuation of our study, we extended our simulation design to include hidden relatedness. Further, in order to test the ability of the new adjusted Armitage test to correct for patterns of hidden relatedness as well as hidden stratification, we considered for illustrative purposes a very extreme situation. We simulated large nuclear families consisting of both parents and 8 offspring, the kinship relationships were regarded as hidden and the families come from two distinct non-mixing hidden strata. In particular, we implemented the following simulation algorithm:

1. We simulated genotypes for one causal SNP (of frequency 50 percent in the ancestral population) and 10,999 non-causal SNPs, placing 500 SNPs on each of 22 independently segregating diploid chromosomes. Ancestral allele frequencies for the non-causal SNPs were simulated as uniform (0.1, 0.9) and the Balding Nichols model was again used to provide the present-day allele frequencies in the two subpopulations, with F = 0.3 relative to the ancestral population.
2. Genotypes for a total of 460 subjects from 46 nuclear families with 8 offspring each in which 23 families came from each of the two different populations. We first sampled chromosomes for the parents and we then computed offspring genotypes assuming Mendelian inheritance.
3. We simulated disease status as a binary variable under a logistic model with an OR of disease = 3 per copy of the causal allele with the background prevalence of disease equal to 10 percent in one population and 35 percent in the other.

In this simulation we computed two versions of the new test and two versions of the principal components test as well as the unadjusted Armitage test and the genomic control method. For the new test we considered two kinship matrices, the empirically estimated one (as described above) and the true kinship matrix taking account of both hidden stratification and the hidden relatedness of the simulated subjects. The true K matrix can readily be shown to be equal to a matrix having within-family block diagonal (10×10) sub-matrices of the form 
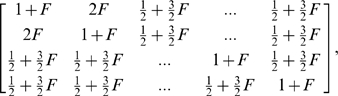
where all other elements equal 2*F* for subjects within the same population or 0 for subjects from different populations. With *F = 0.3* we find, numerically, that the eigenvalues of the true K matrix (size 460×460) have their largest two values equal to 96.32, the next 44 elements equal to 4.32, another 46 values equal to 0.8, 322 values equal to 0.40, and the remaining 46 values equal to 0.0740. Based on this pattern of the eigenvalues in the principal components analysis, we considered using either just the 2 leading eigenvectors (accounting for the marked stratification between populations, and which will clearly be strongly significant as predictors of disease status) or all 48 eigenvectors with eigenvalues ≥0.8 as adjustment variables (in an effort to capture the eigenvectors associated with the relatedness of family members as well).

## Results

In the first two simulations (one with and one without differential genotyping error) we compared the performance of the new test against three commonly used approaches, the unadjusted Armitage test, genomic control and principal components. In the principal components analysis the first 10 eigenvectors are easily found to be related to population substructure and the 11^th^ to differential genotyping error (for the second of the two simulations). In order to capture this overall structure we used a total of 14 eigenvectors in all calculations using the PC method – while the number of eigenvectors actually used was chosen somewhat arbitrarily, the results described below are retained so long as at least 11 eigenvectors are consistently included. Our results show that all eigenvectors except the last 4 were always strongly significant predictors of disease status and the 11^th^ was always significant in the second scenario – thus we have added (as eigenvectors 12, 13, and 14) three principal components which are not generally related to disease status, dropping them would have little effect on the results below.

**Figure 1:**
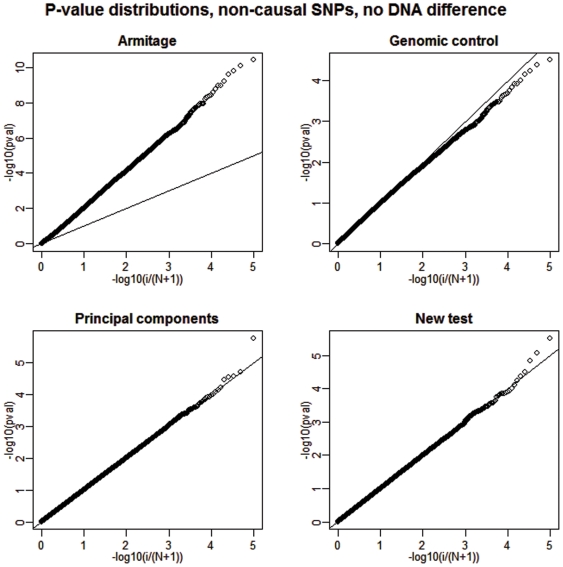
Deviations from uniformity under the null, no DNA differentiation. We show a plot of the distribution of the p-values of all non-causal SNPs for each of the four competing tests when DNA differentiation between cases and controls is absent. The straight broken line represents Uniform (0,1) distribution.

**Figure 2:**
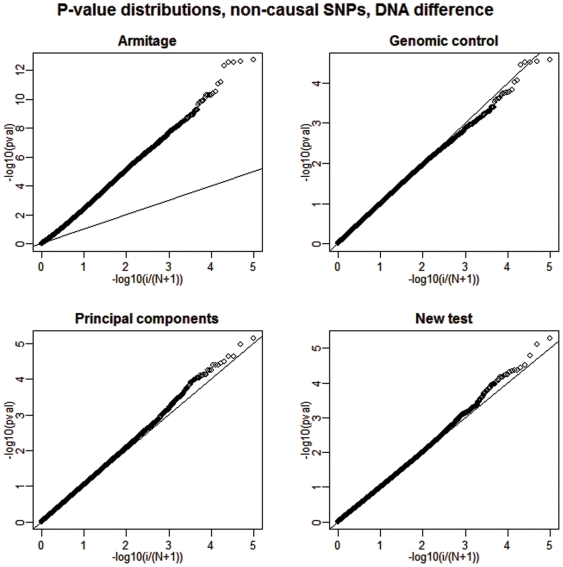
Deviations from uniformity under the null, 1% DNA differentiation. We show a plot of the distribution of the p-values of all non-causal SNPs for each of the four competing tests when 1% DNA differentiation between cases and controls is present. The straight broken line represents Uniform (0,1) distribution.

First, we show the behavior of the type I error rates. A summary of our results from the analysis of 100 simulated datasets under the scenario of the described hidden stratification is presented in Table 1. Figures 1 and 2 give results from a single but representative simulation.

**Table 1:** Empirical Type I error rates for all tests: with hidden stratification.

	Armitage	GC	PC	New test
No DNA difference^a^	0.255	0.048	0.052	0.050
1% DNA difference^a^	0.261	0.048	0.053	0.050
No DNA difference^b^	0.01129	0.00005	0.00009	0.00008
1% DNA difference^b^	0.02496	0.00007	0.00019	0.00012

a nominal α = 0.05.

b nominal α = 0.0001.

As expected, there is a large inflation of false positive outcomes for the Armitage test due to lack of adjustment for the existing population structure or over-dispersion due to genotyping error. The genomic control and principal components approaches do a reasonably precise correction and exhibit almost nominal levels of false positives. However, for the new test the type I error rates appear to be slightly better.

**Figure 3:**
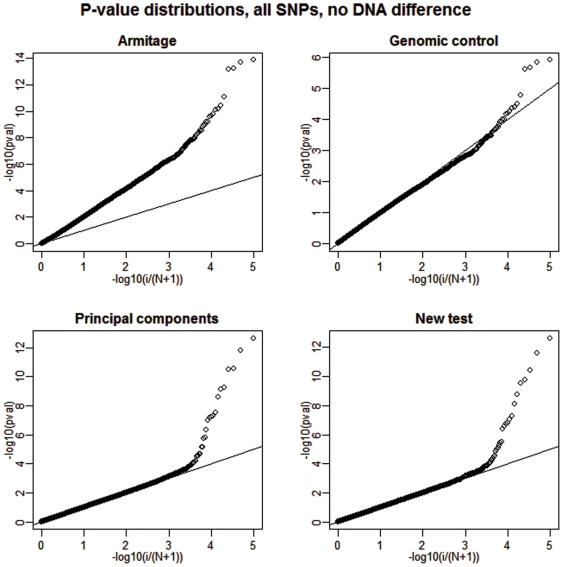
Deviations from uniformity under the alternative, no DNA differentiation. We show a plot of the distribution of the p-values of all SNPs (20 of them are causal) for each of the four competing tests when DNA differentiation between cases and controls is absent. The straight broken line represents Uniform (0,1) distribution.

**Figure 4:**
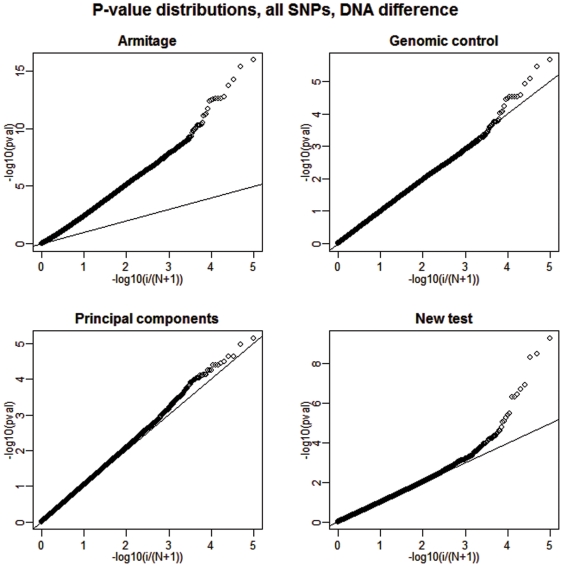
Deviations from uniformity under the alternative, 1% DNA differentiation. We show a plot of the distribution of the p-values of all SNPs (20 of them are causal) for each of the four competing tests when 1% DNA differentiation between cases and controls is present. The straight broken line represents Uniform (0,1) distribution.

Next, we present a summary of our results regarding the power of the analyzed tests in Table 2. The empirical power is calculated as the fraction of the 20 causal SNPs that were detected at two different significance levels and over all 100 replications. See Figures 3 and 4 for a graphical depiction of a single but representative simulation run.

**Table 2:** Power comparison among all tests: with hidden stratification.

	Armitage	GC	PC	New test
No DNA difference^a^	0.55	0.43	0.65	0.66
1% DNA difference^a^	0.75	0.46	0	0.40
No DNA difference^b^	0.90	0.40	0.91	0.95
1% DNA difference^b^	0.75	0.41	0	0.65

a α = 0.05.10^−5^.

b α = 0.0001.

Since we are only interested in the power of tests with close to nominal type I error rates, the results for the Armitage test are inconsequential. It is clear that when DNA preparation-related allele frequency differences between cases and controls are not present, the new test achieves power performance that is only slightly better (0–5%) than the one achieved by the principal component approach. However, when such a DNA difference exists, the new test shows a clear performance advantage by attaining a power gain as large as 58% over the second best testing option. In contrast, the principal components strategy is impeded by the fact that one of the eigenvectors (the 11^th^) is highly correlated with the case-control status variable. Therefore, the inclusion of such eigenvector in the subsequent genotype adjustment completely obliterates the power of this method to detect any remaining systematic differences between the allele frequencies of the cases and the controls. Specifically, in our simulation the 11th eigenvector was highly correlated with case control status (average 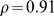). Interestingly, on average, the Armitage test is no more powerful than the new adjusted version despite the excessive over-dispersion under the null.

Simulations results under the scenario of hidden stratification and hidden relatedness regarding the observed false positive error rates of the methods described above are summarized in Table 3. It is clear that in this example the unadjusted Armitage tests and the genomic control method perform very poorly. The statistics of the first are predictably highly over-dispersed while the second approach employs a correction factor that is much too large for the adjustment of the tail areas of the test. Specifically, by manipulating the simulation scenarios (data not shown) we found that the failure of the genomic control method was due to the severe population stratification simulated here rather than to the relatedness between families; the GC method would improve its behavior considerably if stratification was not present. The two methods utilizing principal components behave much better than genomic control with respect to type 1 error rate, although they are consistently between 1.5–3 times higher than the nominal type 1 error rates considered. When either the true or empirically estimated K matrix is used in the adjusted Armitage test, the observed type 1 error rates are very close to their nominal rates. Note that each of the 100 simulations involved 10,999 non-causal markers so that that each observed false positive probability is based on ∼1.1 million simulated SNPs and is therefore estimated very accurately.

**Table 3:** Observed type 1 error rates: with both hidden stratification and hidden relatedness between subjects.

	Armitage	GC	PC (48)	PC (2)	K	True K
P<0.05	.455	.023	.067	0.079	0.051	.051
P<0.01	.337	0.0005	0.016	0.021	0.010	0.010
P<0.005	.300	0.00015	0.0087	0.0116	0.0054	0.0055
P<0.001	0.23	1.44×10^−5^	0.0021	0.0032	0.0012	0.0013

Next, we present a summary of our results regarding the power of the analyzed tests. Table 4 shows the power for detecting true positive associations for the two nearly unbiased tests, namely, the two versions of the adjusted Armitage test. It is clear there is a moderate (between 7–14%) loss of power due to the use of the estimated rather than the true kinship matrix.

**Table 4:** Observed power to detect the true positive marker: with both hidden stratification and hidden relatedness between subjects.

Significance Criteria	K	True K
P<0.01	0.86	0.93
P<0.005	0.79	0.91
P<0.001	0.64	0.78
P<0.0001	0.46	0.60
P<0.00005	0.42	0.54

## Discussion

In this work we propose a new test for association in genome-wide association scans with case-control data. While this test is clearly related to that of Bourgain et al [13], it appears to be a novel suggestion that empirically estimated kinship matrices can be used in a similar statistical approach. Both the principal components and the new methods can be thought of as adjusted Armitage tests. The former adjusts for a selected set of eigenvectors while the latter adjusts the test by assuming a simple and apparently effective model for the variance of the vectors of genotypes that takes into account the pairwise relatedness.

In part, we have focused on a scenario that showcases a deficiency of the principal components approach: when there are small differential genotype differences between cases and controls which we consider to be due to DNA preparation dissimilarities. One of our simulations highlights an example of overcorrection that can occur with the principal components approach. In reality, the DNA preparation-related errors can explain only approximately 1 percent differences in allele frequencies between cases and controls but with the principal components method even very large differences (for the causal SNPs) are attributed to “hidden structure”. Because the new method (similar to genomic control) uses the empirical variability of the SNPs to estimate features of the dispersion of the SNPs, this overcorrection does not occur. The genomic control method assumes a common factor of overdispersion for all SNPs, whereas the new method assumes a common overdispersion matrix 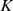 for all SNPs. Thus, the new method may be regarded as an extension of the original genomic control idea that seems to correct for both overdispersion and hidden structure.

We verified our conclusions by studying the performance of the new test in three scenarios: a highly stratified population, a stratified population with additional difference in allele frequencies between cases and controls and a complex population consisting of closely related study participants sampled from two different non-mixing groups. Regarding the first scenario, our results show that the new test and the principal components approach possess desirable type I error rates and offer the best performance with a 0–5% power advantage for the new test. In the second scenario, we conclude that the new test has outstanding properties compared to the other approaches considered. In the third scenario, we show the promising performance of the proposed test for dealing with hidden relatedness. Some loss of power was observed in this scenario due to using an estimated rather than a true kinship matrix, this may be due to using only 11,000 markers (and not 100,000 as in the first two simulations) where we found little differences using the true or estimated **K** (data not shown). Genome-wide association studies of course involve even more markers (from 500,000 to 1 million) which may alleviate this problem further or completely.

The issue of overcorrection for laboratory errors possible with the principal components method has been noted by Price et al. in supplementary materials provided online for their 2006 article. They specifically point out in a section on “assay effects” that eigenvectors can align with plate differences and that if these effects differ between cases and controls (because of plate layout) naïve application of the principal components method can lead to severe power loss. Their solution of removing SNPs with higher than normal missingness is certainly reasonable but further work with real or simulated data illustrating such plate effects would help to determine if our test would still perform better than the principal components methods after removal of the most obvious genotyping problems. In our example with differential genotyping error we simulated a situation that lead to a very sharp difference between cases and controls on one of the leading eigenvectors (#11). It is clear that this would easily be detected and “not believed” and therefore attributed to differential genotyping error. However does one then drop analysis of the data altogether? If the only problem was differential genotyping error then the genomic control method would work very well because for any individual SNP the difference between cases and controls would be very small (and would not swamp differences for a strong causal SNP). However, if there is also hidden stratification, the genomic control method tends to lose power (because it does not “reorder” associations). We are providing a way forward in cases where both problems (genotyping differences and hidden stratification) are present. It is possible that a combination of GC + principal components (dropping the offending eigenvector but not other ones less strongly related to case control status) would work in the example simulated. However, this approach would be contradictory to the conventional wisdom that one should drop principal components that are NOT related to case-control status, rather than principal components that are so related!

Going beyond the issue of DNA preparation errors, as other authors have noted, in the genome-wide association settings we have enough SNPs to be able to detect small differences in ancestry. With the increasingly large amounts of publicly available genome-wide SNP data it is worth considering the various methods for correction for hidden stratification in the context of studies that only genotype disease cases and which rely upon the controls from other studies to complete the case control analysis. For instance, a study in which all controls were to come from Scandinavia but all cases were from other European regions would have eigenvectors highly correlated with descent and hence with case-control status. Thus, a naive application of the PC method will be subject to severe loss of power since this would be analogous to the DNA preparation-related differences scenario that we simulated. Again, the problem with the principal components approach is that it tends to overcorrect for small differences due to slightly different ancestry between cases and controls, differences which could be very small compared to allele frequency differences for a strongly causal SNP. Further work on this problem is clearly warranted given the increasing availability of whole genome SNP data for “normal” subjects.

Our estimation of **K** in the simulations used “unlinked” markers (i.e. markers that were independent conditional both upon the allele frequencies simulated in the Balding-Nichols model and upon the genotyping error). The inclusion of markers illustrating LD would tend to increase the variability of the estimates of the kinship matrix but we suggest (as does Price et al., see supplementary materials) that if most pairs of markers are not linked to each other (as in a genome wide study) that all markers can be used to estimate kinship.

We used 100,000 SNPs in our first two simulations. While this is less than a typical genome-wide association study, it is more than what typically would be genotyped in the 2nd stage of a multistage study which would also require correction for ancestry differences between cases and controls. Our third simulation showed some loss in power when a kinship matrix estimated using 11,000 SNPs was used (relative to using the true **K** matrix). While this was an especially complicated simulated study (with both very close relations between subjects as well as severe hidden stratification), the third simulation clearly raises the question of whether or not a typical stage 2 of a genome–wide association scan will genotype enough markers to adequately estimate **K**. Additional simulations will be needed to settle this important question.

An obvious issue for our approach is the inclusion of other variables in the analysis. These covariates could be related to case-control status either in addition to genetic causes or could be modifiers of genetic causes. Although we have not yet done extensive simulation work to check, we suspect that adding such variables to columns of the matrix **C** described above will allow for the effect of additional variables or potential confounders to be estimated or adjusted for. Interactions between genetic and non-genetic variables could certainly be considered in (an extension of) case/only analyses [14] in which kinship among the cases is now adjusted for in an examination of whether the genotypes of the cases are correlated with potential effect modifiers (i.e. G×E interactions).

### Other observations

As noted in the methods section, some special designs such as sibling-matched case-control studies and parent-offspring trios yield a **K** matrix for which the proposed test produces familiar and appropriate analyses. We are not proposing that this approach be taken in the analysis of such a designed study, but it is interesting to compare the result of the new test with either the principal components or genomic control method. If the only issue to be dealt with is close relatedness between cases and controls from the same family, then our other experience is that the genomic control method works very well and is certainly competitive with our proposal. On the other hand, analysis of either sibling-matched or parent-offspring pairs by the principal components method produces poor results compared to genomic control and the results can swing wildly from severe under-correction to over-correction depending upon the number of eigenvectors chosen for the adjustments. If both hidden population stratification and strong family relationships are present, genomic control will no longer be as effective (as in our 3^rd^ simulation) as the proposed test.

We have pointed out the similarities between this test and that of Bourgain et al. There are several other papers that consider similar use of kinship matrices when pedigrees are known. Slagger and Schaid [15] propose an approach that modifies the variance of the classical Armitage trend test to handle the scenario of sampling related subjects from extended pedigrees. The variance adjustment that accounts for relatedness is done in a fashion very similar to the genomic control variance inflation estimation. This test has been proposed in candidate gene studies and requires estimation of IBD probabilities (done by an implementation of the Lander-Green algorithm via a separate run of the GENEHUNTER software [16]) for each pair of subjects and at each SNP making its natural extension to genome-wide studies computationally intensive. Our method provides a more computationally efficient way while having a simple model-based derivation. Chen and Abecasis [17] propose an approach for detecting association between quantitative traits and genotypes. The method implements a simple model for the mean of the phenotypes (we model the mean of the genotypes). The conceptual similarity to our method can be seen in the imposed model for the variance that reflects the relatedness between each pair of subjects, although again, the emphasis in Chen and Abecasis was upon known pedigree relationships. Lastly, Amin et al. [18] propose a two-stage approach for association between quantitative traits and genotypes that implements a heritability estimation step and SNP testing step that is based on the residuals obtained from step one. While this method uses the same empirical correlation matrix that we employ, it uses this matrix in order to estimate the pedigree structure which is needed for the heritability estimation. Therefore, it is not clear whether the Amin et al. method would be applicable to genetic association studies with population substructure but otherwise little close relatedness between subjects.

### Statistical software

A self-contained R and C++ implementations of the proposed test will soon be available at: www-rcf.usc.edu/∼stram.
